# Cross-cultural differences in implicit learning of chunks versus symmetries

**DOI:** 10.1098/rsos.180469

**Published:** 2018-10-17

**Authors:** Xiaoli Ling, Li Zheng, Xiuyan Guo, Shouxin Li, Shiyu Song, Lining Sun, Zoltan Dienes

**Affiliations:** 1 School of Psychology, Shandong Normal University, Jinan, People's Republic of China; 2 School of Psychology and Cognitive Science and Shanghai Key Laboratory of Magnetic Resonance, East China Normal University, Shanghai, People's Republic of China; 3 Key Laboratory of Brain Functional Genomics, Ministry of Education, Shanghai Key Laboratory of Brain Functional Genomics, East China Normal University, Shanghai, People's Republic of China; 4 Shanghai Key Laboratory of Magnetic Resonance and School of Psychology and Cognitive Science, East China Normal University, Shanghai, People's Republic of China; 5 School of Psychology and Cognitive Science, East China Normal University, Shanghai, People's Republic of China; 6 Department of Educational Psychology, University of Connecticut, Storrs, CT, USA; 7 School of Psychology and Sackler Centre for Consciousness Science, University of Sussex, Brighton, UK

**Keywords:** implicit learning, cross-cultural differences, global/local, chunks, symmetries

## Abstract

Three experiments explore whether knowledge of grammars defining global versus local regularities has an advantage in implicit acquisition and whether this advantage is affected by cultural differences. Participants were asked to listen to and memorize a number of strings of 10 syllables instantiating an inversion (i.e. a global pattern); after the training phase, they were required to judge whether new strings were well formed. In Experiment 1, Western people implicitly acquired the inversion rule defined over the Chinese tones in a similar way as Chinese participants when alternative structures (specifically, chunking and repetition structures) were controlled. In Experiments 2 and 3, we directly pitted knowledge of the inversion (global) against chunk (local) knowledge, and found that Chinese participants had a striking global advantage in implicit learning, which was greater than that of Western participants. Taken together, we show for the first time cross-cultural differences in the type of regularities implicitly acquired.

## 1. Introduction

Implicit learning is the term coined by A.S. Reber [1,2] to describe this process by which people can acquire knowledge of the structure in the environment without awareness. A fundamental issue for the field is what sort of structures can be implicitly learnt. Reber [3] argued that people can implicitly learn abstract rules. By contrast, others have argued that implicit learning may consist only of learning chunks (i.e. bigrams and trigrams that appeared in the string; e.g. the letter string ‘NVJTVJ’ includes the bigram chunks NV, VJ, JT, TV and VJ, and the trigram chunks NVJ, VJT, JTV and TVJ; see [4]) or specific sequences constituting the learned exemplars (e.g. [5,6]). Both sides of the debate have made credible demonstrations that rules (beyond chunks and exemplars) have or have not been learnt in the experimental context ([7–12], e.g. [13]). However, previous studies using finite-state grammars (i.e. structural rules constraining which letters can follow other letters) make it difficult to isolate the contributions of knowledge of rules and chunks, as the structure in the grammars is largely captured by allowable chunks [14]; and demonstrations failing to find implicit learning of other types of rules have often used rules with low prior probability of being relevant for a human learning system (e.g. [7], cf. [15]).

Recently, Jiang *et al.* [16] investigated this issue using supra-finite-state grammars (e.g. cross-serial dependencies), which involved long-distance dependencies and allowed independent manipulation across test items of grammaticality and chunk strength (i.e. the frequency with which bigrams and trigrams in the test strings had occurred in the training set; cf. [17]). The grammar employed by Jiang *et al.* was an inversion rule (i.e. a type of cross-serial dependency) and was defined over the tones with which Chinese syllables are spoken. The rule involves a type of symmetry, and symmetry is *a priori* useful for a perceptual system to be sensitive to (for the computational relevance of symmetry to perception see [18], for evidence for its empirical relevance to implicit learning, e.g. [19–21]). There are four tones in Mandarin Chinese (1–4) indicating flat, rising, falling-rising and falling phonetic characteristics in pitch, respectively. Tones 1 and 2 are categorized into ping (level) tones, while tones 3 and 4 are categorized into ze (oblique) tones. Jiang *et al*. constructed strings in which the tone types (ping or ze) of the first five tonal syllables of a string predicted those of the last five by an inversion relation. In Jiang *et al*.'s paradigm, participants are asked to silently repeat a number of strings of 10 syllables instantiating this regularity. After the training phase, participants are then informed that the strings followed a set of rules and required to judge new strings as well formed or not. The results showed that, controlling both chunking and repetition structure, participants can implicitly learn abstract rules employing features of long-distance dependencies.

A growing body of artificial grammar-learning literature using finite-state grammars has attempted to cross classify materials according to rules (probably reflecting repetition pattern) and chunks [22–26]. However, in the Jiang *et al.*'s study, the materials were designed to control the chunks and the repetition patterns found in strings, which allowed researchers to investigate the implicit learning of only rules (non-local dependencies) but not explore the implicit learning of both rules and chunk knowledge. It leaves open question which type of knowledge (i.e. rules or chunks) has an advantage in implicit acquisition, when chunks and regularities other than chunks are both presented. The inversion rule used in Jiang *et al.* [16] involved long-distance dependencies and thus allowed us to unconfound knowledge about grammaticality from knowledge about chunks of adjacent elements. Therefore, in the current study, we directly pitted knowledge of non-local dependencies against chunk knowledge to further explore which type of knowledge has an advantage in implicit acquisition by using the inversion rule used in Jiang *et al*.

Participants in Jiang *et al.* [16] were all native Chinese speakers. This leaves open the question of the extent to which an inversion over language tones can be learnt by other language and cultural groups, especially Westerners. Research on cross-cultural differences in cognitive processes has demonstrated that Asians use a more global processing style, being especially sensitive to contextual information in conscious processes (e.g. perceptual judgement and memory), whereas Westerners take a more analytic approach, preferentially attending to object-related information [27–29]. Recently, Kiyokawa *et al.* [30] investigated whether these biases also apply to unconscious knowledge using finite-state grammars with GLOCAL strings, which are chains of compound large letters made out of small letters (i.e. local letters). In their study, GLOCAL strings were generated following two different artificial grammars at the level of large letters and small letters, respectively. Participants were asked to memorize the strings (without being directed to a specific level) in the training phase. The results showed that Japanese classified test strings corresponding to the global rather than local grammar more accurately, whereas English performed similarly on both grammars even when the acquired knowledge was unconscious, indicating that Japanese showed a global advantage even when the knowledge was obtained unconsciously. In this case, the regularities at both the global and local level were standard finite-state grammars; it was the letters that were presented globally or locally (see also [31], for similar results with a different implicit learning task). The finding leaves open whether there could be cross-cultural differences in the sort of rules learnt. Therefore, we will explore for the first time which type of knowledge (i.e. global symmetries or more local chunks) has an advantage in implicit acquisition and whether this advantage will be affected by cultural differences.

**Figure 1.**
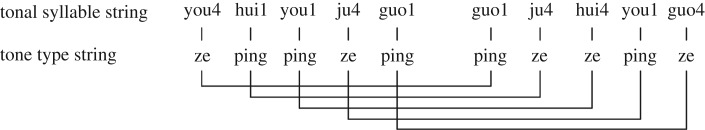
An example of grammatical strings in Experiment 1. As in Jiang *et al.*, the inversion rule used in Experiment 1 was defined over the tones of Chinese language (with respect to the four tones, where tones 1 and 2 are traditionally categorized as ping tones, while tones 3 and 4 are categorized as ze tones). Ten tonal syllables constitute a string and the first five tonal syllables formed the prime, while the last five tonal syllables formed the inversion, which was created by a mapping between the tone type of the tonal syllables (i.e. ping was mapped to ze). For example, if the tone type of the first tonal syllable was ping, then the tone type of the sixth tonal syllable was ze, and if the type of the second tonal syllable was ping, the tone type of the seventh tonal syllable was ze, and so on.

In the current study, three experiments were conducted to explore these issues by using the Jiang *et al.* [16] artificial Chinese poetry paradigm. Like Jiang *et al*., we presented participants with strings of 10 tonal syllables where the tone types (Experiments 1 and 2) or syllables (Experiment 3) of the first five tonal syllables predicted the following inversion (see figures 1 and 4). In the training phase, participants were requested to repeat back a number of Chinese tonal syllable strings instantiating an inversion; in the test phase, they responded to new strings either following or violating the same rules of construction as the training strings. Experiment 1 investigates whether Westerners could implicitly learn the inversion rule made out of Chinese tonal syllables in a similar way as Chinese people, when controlling the chunks and the repetition patterns found in strings. Experiments 2 and 3 aimed to explore whether there is a priority of different types of structures (i.e. symmetries versus chunks) people implicitly learnt and whether this process will be affected by cross-cultural differences, by orthogonally manipulating grammaticality and chunk strength for test strings. The theory that Easterners rather than Westerners are relatively more sensitive to global rather than local structures predicts that Chinese would classify more accurately test strings corresponding to the inversion rule rather than adjacent chunks to a greater extent than Westerners, who might even show a reverse preference. In summary, in contrast to previous work, the current experiments do not address the role of selective attention to global or local elements in implicit learning; but rather to the type of regularity that is best implicitly learned when the elements themselves are well attended.

The implicit nature of the knowledge was determined by using the structural knowledge attributions of Dienes & Scott [32]. They distinguished judgement knowledge from structural knowledge: judgement knowledge is the knowledge directly expressed in the judgement that a string is grammatical (e.g. the knowledge that ‘this string is grammatical’); structural knowledge is the knowledge that enabled that judgement (e.g. the knowledge that ‘symmetry makes a string grammatical’). Participants indicate for each grammaticality judgement whether it was based on random guessing (the participant judges that there was no knowledge), intuition (the participant has no idea why their answer is right, but they think it is) and memory (they recollected or failed to recollect a similar training sequence) or rules they could state if asked. Guess and intuition responses are taken to indicate unconscious structural knowledge, while memory and rule responses are combined as indicators of conscious structural knowledge (e.g. [10,16,30,33–38]).

## 2. Experiment 1

Experiment 1 aimed to show directly whether Western people could implicitly learn the inversion rule made out of the Chinese tonal syllables as Chinese people can, when controlling the chunks and the repetition patterns found in strings by using the Jiang *et al.* [16] artificial Chinese poetry paradigm. Following Jiang *et al.*, in this experiment, the rule was defined over the tones with which Chinese syllables were spoken. We presented participants with strings of 10 tonal syllables where the tone types (pings or zes) of the previous five tonal syllables of a string predicted those of the last five by an inversion relation (figure 1). That is, ping in the first half maps to ze in the last half in the same corresponding position in the sequence (e.g. if the tone type of the first tonal syllable was ping, then the tone type of the sixth tonal syllable was ze), and ze likewise maps to ping in corresponding positions.

### 2.1. Method

#### 2.1.1. Participants

Seventeen Chinese students (10 females, aged from 18 to 27, *M* = 21.53; s.d. = 2.79) and 17 international Western students (six French, five Italians, one Ukrainian, one German, one British, three Americans; 10 females, aged from 19 to 26, *M* = 22.06; s.d. = 2.14) were recruited from the universities in Shanghai. All of the Chinese participants were born in China. Western participants were all students who came to China for a short-term exchange and learn Chinese. All had acquired mastery of the tonal syllables used in Experiment 1 as we show below (in the pre-test). Participants from the two groups were matched on age and gender. All the participants received course credits or monetary compensation for their participation. None of them had a history of hearing difficulties.

#### 2.1.2. Materials

In this experiment, eight Chinese tonal syllables were selected (i.e. guo1, guo4, you1, you4, hui1, hui4, ju1 and ju4) and pronounced using a Chinese pronunciation software (Xunfei interphonic 2.30, sampling rate = 22.05 kHz) with a female voice. Each of the tonal syllables lasted for 400 ms. The tone type of four of the tonal syllables belongs to ‘ping’: guo1, you1, hui1 and ju1; the tone type of other four tonal syllables belongs to ‘ze’: guo4, you4, hui4 and ju4. Ten tonal syllables constitute a string in which the tone types (pings or zes) of the first half tonal syllables predicted that of the last half according to the inversion rule, resulting to five ping–ze pairs (figure 1). In the current study, ping–ze pairs refer to tone1–tone4 pairs. Each tonal syllable string were created by concatenating these individual sound files and adding 600 ms silences between the fifth and sixth tonal syllables to create a perceptual gap between the first half of the string and its inversion in the final half (cf. [16,39]).

Thirty-two grammatical tone-type strings were generated based on the rule of inversion, 16 of which served as training strings, and the rest were used as test strings. Each training tone-type string generated three tonal syllable strings, resulting in 48 training tonal syllable strings in all (e.g. grammatical tone-type string ‘ze ping ping ze ping – ping ze ze ping ze’ can generate tonal syllable strings ‘hui4 ju1 guo1 you4 ju1 – you1 hui4 guo4 ju1 hui4’, ‘hui4 guo1 ju1 guo4 you1 – hui1 you4 hui4 ju1 guo4’, ‘you4 hui1 you1 ju4 guo1 – guo1 ju4 hui4 you1 guo4’). The test set contained 16 grammatical tone-type strings and 16 ungrammatical tone-type strings which were constructed by violating the inversion rule in any two of five ping–ze pairs of 16 grammatical strings in the test. Each test tone-type string was shown twice with different tonal syllables, resulting in 64 test tonal syllable strings in all. None of the tonal syllable strings had a clear semantic interpretation.

Furthermore, mean feature frequency (MFF), anchor associative chunk strength (AACS) and global associative chunk strength (GACS) were balanced across grammatical and ungrammatical test tone-type strings. MFF for each test tone-type string was defined as the average number of times each tone type appeared in the training phase in each of the 10 positions. The GACS was calculated for each test string by averaging the frequency with which bigrams and trigrams contained in a test string occurred in the training set. AACS was defined as the frequency with which tone-type bigrams and trigrams occurred in the beginning and ending positions. In addition, the MFF, AACS and GACS in terms of syllables (e.g. the syllable string of the tonal syllable string ‘hui4 guo1 hui4 you1 ju1 – you1 ju4 hui1 guo4 you4’ was ‘hui guo hui you ju – you ju hui guo you’) were also balanced between grammatical and ungrammatical strings. Grammatical and ungrammatical strings were also balanced along the same dimensions in terms of tonal syllables (table 1, all strings together with their MFFs, AACSs and GACSs, see electronic supplementary material, table S1). Repetition structures of grammatical and ungrammatical test tone-type strings were controlled. Repetition structure reflects whether any element is the same as any other element in the string; for example, the repetition structure of ‘ze ping ping ze ping’ or ‘ping ze ze ping ze’ is 12212 [40]. None of the test strings had the same repetition structures, in terms of tone types or syllables, as any of the training strings.

**Table 1.** Mean MFF, GACS and AACS for grammatical and ungrammatical strings in terms of tone types (ping and ze), syllables and tonal syllables (*M* ± s.d.). G, grammatical; UG, ungrammatical; MFF, mean feature frequency; GACS, global associative chunk strength; AACS, anchor associative chunk strength.

		MFF	GACS	AACS
tone type	G	720.00 ± 0.00	223.31 ± 5.64	25.88 ± 2.29
	UG	720.00 ± 0.00	222.21 ± 8.76	25.73 ± 1.39
syllables	G	360.00 ± 0.00	50.48 ± 2.43	5.72 ± 1.27
	UG	360.00 ± 0.00	50.55 ± 2.34	5.72 ± 1.57
tonal syllables	G	180.02 ± 1.00	12.08 ± 1.83	1.22 ± 0.91
	UG	179.82 ± 1.06	11.76 ± 1.53	1.38 ± 0.76

#### 2.1.3. Procedure

Before the training phase, participants performed a pre-test (i.e. a tonal syllable discernment task). In the task, participants listened to two tonal syllables which were randomly selected from eight tonal syllables and then judged whether the two tonal syllables were the same or not by pressing ‘F’ or ‘J’ key as accurately as possible. There were in total 128 pairs of tonal syllables, which consisted of 64 pairs of tonal syllable repeated two times in a random order.

During the training phase, 48 grammatical tonal syllable strings were played to participants one at a time. Each trial began with the presentation of a 500 ms warning tone and a 500 ms blank, followed by a training tonal syllable string and a 5000 ms blank. Participants were told to listen to each tonal syllable string carefully and silently repeat it during the 5000 ms delay before the next trial. The presentation of these 48 strings was then repeated a second time and third time in a random order, resulting in 144 strings in all.

After the training phase, participants were informed that each of the strings that they heard in the training phase was generated based on a specific rule and were asked whether they detected the rule. Then, they were required to listen to 64 new tonal syllable strings which were presented in a random order and told that half of them followed the same rule as the strings in the training phase while the other half of them did not. For each test string, the participants were required to judge whether the given string was legal or not. To assess the conscious status of the acquired knowledge, we used the method of structural knowledge attributions introduced by Dienes & Scott [32]. This method allowed us to systematically establish the conscious status of the acquired knowledge in a sensitive way. Specifically, after each classification decision, participants were asked to give the basis for their decision by choosing one of the following four structural knowledge attributions [41]: ‘Guess’ indicated that you have no idea whether the given string fits the rule or not and the judgement could just as well have been based on a toss of a coin; ‘Intuition’ indicated that your judgement was based on a hunch or feeling that could not be explicated further, i.e. there was confidence in the judgement but you had no idea why the judgement was right; ‘Memory’ indicated that the judgement was based on a recollection (or a failure to recollect); ‘Rule’ indicated that the judgement was based on a rule that could be stated if asked. Unconscious structural knowledge was indicated by the attributions of guess and intuition, while conscious structural knowledge by those of memory and rule (e.g. [10,16,30,33–38]).

### 2.2. Results

Bayes factors (*B*) were used to assess strength of evidence for the alternative hypothesis, H1, over the null, H0 ([42], for the use of Bayes factors in implicit cognition research, see [43,44]). A *B* of above 3 indicates substantial evidence for H1 over H0 and below 1/3 substantial evidence for the H0 over Hl. All Bayes factors, *B*, reported here represent the evidence for H1 relative to H0; to find the evidence for H0 relative to H1, take 1/*B*. *B*s between 3 and 1/3 indicate data insensitivity (cf. [45], see [46]). Here, *B*_H(0, *x*)_ refers to a Bayes factor in which the predictions of H1 were modelled as a half-normal distribution with an s.d. of *x* (see [46]); the half-normal can be used when a theory makes a directional prediction where *x* scales the size of effect that could be expected (so *x* can be chosen from, for example, relevant past studies). Jiang *et al.* [16] and Li *et al.* [47], using similar stimuli, found differences of the order of 5% in classification accuracy between conditions; thus, for classification performance, we use *B*_H(0, 5%)_ for all tests. With these assumptions for modelling H1, as it happened, where an effect yielded a *p-*value of about 0.05, the Bayes factor was about 3, though there is no guarantee of such a correspondence between *B* and *p-*values, which are not related monotonically [43,48]. We will interpret all effects with respect to the Bayes factors.

#### 2.2.1. Pre-test

The proportions of correct response for Chinese and Western groups in the pre-test were 0.99 (s.d. = 0.01) and 0.98 (s.d. = 0.02), respectively. The evidence was insensitive for whether or not there was a difference between Chinese and Western groups, *t*_32_ = 1.52, *p* = 0.139, *d* = 0.52, *B*_H(0, 5%)_ = 0.53.

#### 2.2.2. Test

The classification accuracy was calculated by (*N*_C_ + 0.5)/(*N* + 1) (*N*_C_ being the number of correct responses and *N* being the total number of responses). In Bayesian terms, adding 0.5 to the number correct and the number incorrect implements a unit information prior [49], corresponding to the belief that with approximately 95% probability classification accuracy lies between 0.05 and 0.95. Such a prior, though vague, adds some information and can increase the accuracy of estimates when data are limited [50,51].

##### 2.2.2.1. Overall classification accuracy

**Figure 2.**
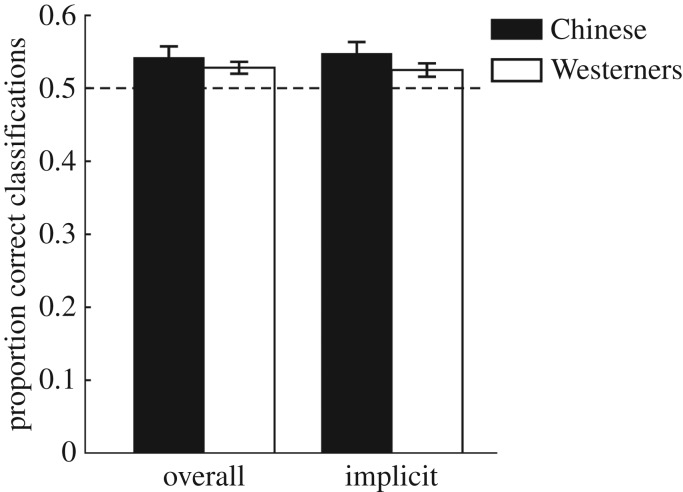
Overall performance (left) and proportion correct classifications by implicit attributions (right) in Experiment 1. Error bars indicate standard error of the mean.

The overall classification performance for Chinese and Western groups were 0.54 (s.d. = 0.06) and 0.53 (s.d. = 0.03), respectively. The performance for each group was better than chance (Chinese: *t*_16_ = 2.88, *p* = 0.011, *d* = 0.70, *B*_H(0, 5%)_ = 15.17; Western: *t*_16_ = 3.49, *p* = 0.003, *d* = 0.85, *B*_H(0, 5%)_ = 53.76, see the left-hand side of figure 2). The evidence was not sensitive for whether or not there was a difference between the two groups (*t*_32_ = 0.86, *p* > 0.250, *d* = 0.29, *B*_H(0, 5%)_ = 0.71).

##### 2.2.2.2. Unconscious structural knowledge

The attributions of guess and intuition were combined as indicators of unconscious structural knowledge (implicit attributions), whereas those of memory and rule were combined as indicators of conscious structural knowledge (explicit attributions) (see [52], for a review of this method). The response proportions and the associated accuracy of each attribution of each group are shown in table 2. Considering that only nine Chinese and 13 Western participants chose explicit attributions in a few of test strings (mean proportion of explicit attributions was *M* = 0.09, s.d. = 0.14), we analysed the classification accuracy based on only implicit attributions. For implicit attributions, the classification of each of Chinese and Western groups was again above chance (Chinese: *t*_16_ = 3.15, *p* = 0.006, *d* = 0.76, *B*_H(0, 5%)_ = 29.17; Western: *t*_16_ = 2.71, *p* = 0.015, *d* = 0.66, *B*_H(0, 5%)_ = 8.06, see the right-hand side of figure 2). The evidence was not sensitive as to whether or not there was a difference between the two groups (*t*_32_ = 1.31, *p* = 0.202, *d* = 0.45, *B*_H(0, 5%)_ = 1.28).

**Table 2.** Response proportions and associated accuracy of each attribution for the Chinese and Western groups in Experiment 1 (*M* ± s.d.).

		implicit attribution		explicit attribution	
		guess	intuition	memory	rule
response proportions	Chinese	0.22 ± 0.16	0.74 ± 0.16	0.04 ± 0.09	0.00 ± 0.00
	Western	0.43 ± 0.28	0.43 ± 0.22	0.12 ± 0.14	0.03 ± 0.06
accuracy	Chinese	0.50 ± 0.16	0.55 ± 0.08	0.48 ± 0.20	
	Western	0.52 ± 0.11	0.54 ± 0.06	0.45 ± 0.16	0.71 ± 0.17

### 2.3. Discussion

The purpose of Experiment 1 was to explore whether Western people could implicitly learn the inversion rule instantiated in Chinese tonal syllables. We showed, using artificial poetry to control both n-gram structure and repetition patterns, that Western people implicitly acquired the inversion rule made out of the Chinese tonal syllables in a similar way as Chinese participants. The data were not sensitive enough to say whether there was a difference in the magnitude of this learning between the groups. Nonetheless, these findings extend Jiang *et al.* [16] and Li *et al.* [47], implying that the tonal inversion paradigm can be effectively applied to implicit learning research beyond native Chinese participants. Importantly, the present results provide further evidence that implicit learning can go beyond the learning of chunks of adjacent elements, challenging fragment models of implicit learning that assume that implicit learning only involves learning chunks (see [53]) or exemplars constituted by chunks (cf. [54,55]). However, in Experiment 1, we controlled both chunks and repetition patterns, therefore leaving open the question as to whether there is a priority for processing different types of structures (i.e. symmetries and chunks) when both structures are simultaneously present; and whether this process is affected by cross-cultural differences. Experiment 2 addressed this issue by exploring symmetry and chunk learning in the same experiment.

Considering that only 22 participants chose explicit attributions in only a few test strings, we analysed the classification accuracy based on only implicit attributions. However, it should be noted that there was no evidence for reported response accuracy in memory attribution being higher than chance (0.5), which would be expected if the participants had explicit knowledge. These results are consistent with the study by Jiang *et al*. [16], which adopted the same experimental paradigm. Sensitive evidence could not be expected with few trials and the large s.d. of responses for memory attributions (mean response proportion was *M* = 0.08, s.d. = 0.12 in the current study; mean response proportion was *M* = 0.19, s.d. = 0.15 in the study by Jiang *et al.*).

## 3. Experiment 2

The aims of Experiment 2 were twofold. The first aim was to identify the type of knowledge that participants acquired while pitting the inversion rule against chunk knowledge. The second aim was to explore cross-cultural differences in implicit learning. In the present experiment, unlike Experiment 1, we orthogonally manipulated grammaticality and chunk strength for test strings. Thus, there were four types of test strings: grammatical items of high chunk strength (GH), grammatical items of low chunk strength (GL), ungrammatical items of high chunk strength (UGH) and ungrammatical items of low chunk strength (UGL; all the chunks of Experiment 1 were of low chunk strength). According to the findings that Asians show a global advantage even when the knowledge is unconscious [30] and the fact that inversion is a property of a whole string, whereas chunks can be determined locally [56], there should be cultural differences in the priority of implicitly learning different types of structures simultaneously present: Chinese should classify more accurately test strings corresponding to the inversion rule rather than adjacent chunks, compared to Westerners, who might even show a reverse or neutral bias.

### 3.1. Method

#### 3.1.1. Participants

Seventeen Chinese students (11 females, aged from 20 to 38, *M* = 23.41; s.d. = 5.30) and 18 international Western students (one Ukrainian, nine Italian, one Brazilian, two Australians, three Americans, one Latvian, one Russian; 11 females, aged from 20 to 36, *M* = 24.28; s.d. = 4.73) from the university community participated in this experiment. All Chinese participants were born in China. All Western participants were students who came to China for a short-term exchange and learn Chinese. All had acquired mastery of the tonal syllables used in Experiment 2 to an equivalent level as Chinese, as we show below (in the pre-test). Participants from the two groups were matched on age and gender. Participants received course credits or monetary compensation for their participation. None had a history of hearing difficulties.

#### 3.1.2. Materials

The eight Chinese tonal syllables (i.e. guo1, guo4, you1, you4, hui1, hui4, ju1 and ju4) and grammatical tone-type strings used in Experiment 2 were identical with those used in Experiment 1. To match the goal of the current experiment, 48 new training tonal syllable strings and 64 new test tonal syllable strings were generated based on the tone-type strings. Half of the grammatical and ungrammatical strings were allocated to high chunk strength strings, whereas the remaining strings of each set was allocated to low chunk strength strings based on the GACS in terms of syllables. Thus, there were four types of test tonal syllable strings: GH, GL, UGH and UGL. Sixty-four test tonal syllable strings were selected from the pool of possible tonal syllable strings created by the test tone-type strings to match this goal and each string-type (GH, GL, UGH and UGL) consisted of 16 test items. High chunk and low chunk strings differed substantially in GACS in terms of four syllables, while the GACS in terms of syllables were balanced over grammatical and ungrammatical strings (table 3, all strings together with their MFFs, AACSs and GACSs, see electronic supplementary material, table S2). In addition, the MFF and AACS in terms of syllables were also balanced over grammatical and ungrammatical strings. Grammatical and ungrammatical strings were also balanced along the same dimensions in terms of tone type and tonal syllables.

**Table 3.** Mean MFF, GACS and AACS for grammatical and ungrammatical strings in terms of tone type, syllables and tonal syllables (high chunk strength and low chunk strength; *M* ± s.d.). G, grammatical; UG, ungrammatical; H, high chunks; L, low chunks; MFF, mean feature frequency; GACS, global associative chunk strength; AACS, anchor associative chunk strength.

			MFF	GACS	AACS
tone type	G	H	720.00 ± 0.00	233.72 ± 2.31	25.88 ± 2.01
		L	720.00 ± 0.00	233.72 ± 2.31	25.88 ± 2.01
	UG	H	720.00 ± 0.00	234.28 ± 1.39	25.88 ± 2.01
		L	720.00 ± 0.00	234.28 ± 1.39	25.88 ± 2.01
syllables	G	H	359.63 ± 0.67	71.84 ± 1.88	8.48 ± 2.58
		L	359.94 ± 0.80	56.66 ± 2.58	8.16 ± 3.26
	UG	H	360.13 ± 0.68	71.66 ± 2.10	7.27 ± 2.91
		L	359.81 ± 0.69	56.89 ± 2.00	6.56 ± 3.03
tonal syllables	G	H	180.68 ± 3.56	16.38 ± 2.02	1.83 ± 1.50
		L	179.29 ± 5.39	12.07 ± 1.64	1.92 ± 1.25
	UG	H	180.01 ± 4.20	15.40 ± 1.00	1.88 ± 1.10
		L	179.59 ± 5.50	12.63 ± 1.61	1.31 ± 0.89

#### 3.1.3. Procedure

The procedure was identical to that used in Experiment 1, except that participants were required to give the basis for their decision by choosing one of five, instead of four, structural knowledge attributions for each test string [41]: guess, intuition, familiarity, recollection and rule. ‘Familiarity’ indicated that the string seemed familiar or unfamiliar for reasons you could not state.

### 3.2. Results

#### 3.2.1. Pre-test

The proportions of correct responses for Chinese and Western groups in the pre-test were 0.99 (s.d. = 0.02) and 0.99 (s.d. = 0.01), respectively. There was no difference between Chinese and Western groups (*t*_33_ = 0.67, *p* > 0.250, *d* = 0.23, *B*_H(0, 5%)_ = 0.05).

#### 3.2.2. Test

The classification accuracy was calculated by (*N*_C_ + 0.5)/(*N* + 1) (*N*_C_ being the number of correct responses and *N* being the total number of responses), as for Experiment 1.

##### 3.2.2.1. Overall classification accuracy

**Figure 3.**
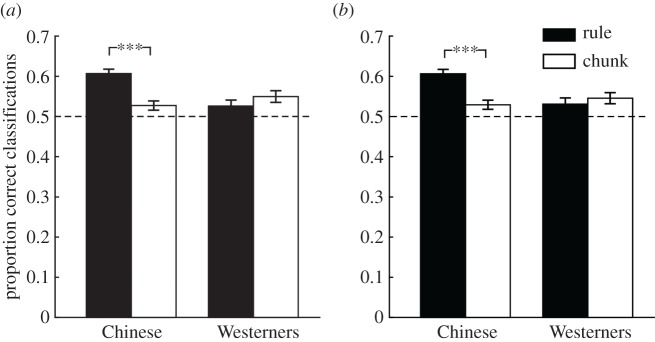
(*a*) Overall performance in Experiment 2. (*b*) Proportion correct classifications by implicit attributions in Experiment 2. ‘Classification performance based on rule’ refers to accuracy on classification decisions based on the ‘rule knowledge’ category (i.e. grammatical versus ungrammatical). ‘Classification performance based on chunk’ refers to accuracy on classification decisions based on the ‘chunk knowledge’ category (i.e. high chunk strength versus low chunk strength). Error bars indicate standard error of the mean (***p < 0.001).

For the Chinese participants, classification performance based on rule and chunks were both above chance (rule: *t*_16_ = 9.71, *p* < 0.001, *d* = 2.36, *B*_H(0, 5%)_ = 18.77 × 10^15^; chunks: *t*_16_ = 2.35, *p* = 0.032, *d* = 0.57, *B*_H(0, 5%)_ = 4.44, figure 3*a*) and classification performance based on rule was greater than that based on chunks (*t*_16_ = 4.55, *p* < 0.001, *d* = 1.10, *B*_H(0, 5%)_ = 1656.81). For the Western participants, classification performance based on chunks was above chance (*t*_17_ = 3.41, *p* = 0.003, *d* = 0.80, *B*_H(0, 5%)_ = 60.99), whereas for rules the evidence was not sensitive as to whether or not classification performance was above chance (*t*_17_ = 1.68, *p* = 0.112, *d* = 0.39, *B*_H(0, 5%)_ = 1.76). There was insensitive evidence as to whether or not there was a chunking advantage (*t*_17_ = 1.38, *p* = 0.185, *d* = 0.33, *B*_H(0, 5%)_ = 1.28). But crucially, the global advantage of Chinese people was greater than that for Western (*t*_33_ = 4.20, *p* < 0.001, *d* = 1.42, *B*_H(0, 5%)_ = 1072.75).

##### 3.2.2.2. Unconscious structural knowledge

The response proportions of each attribution of each group are shown in table 4 and the associated accuracy of each group are shown in table 5. The attributions of guess, intuition and familiarity were combined as indicators of unconscious structural knowledge (implicit attributions), whereas those of memory and rule were combined as indicators of conscious structural knowledge (explicit attributions) [41]. Considering that only two Chinese and nine Western participants chose explicit attributions in a few of test strings (mean proportion of explicit attributions was *M* = 0.03, s.d. = 0.09), we analysed the classification accuracy based on only the implicit attributions.

**Table 4.** Response proportions of each attribution for the Chinese and Western groups in Experiment 2 (*M* ± s.d.).

	implicit attribution			explicit attribution	
	guess	intuition	familiarity	recollection	rule
Chinese	0.28 ± 0.18	0.58 ± 0.23	0.20 ± 0.23	0.03 ± 0.02	0.00 ± 0.00
Western	0.18 ± 0.14	0.45 ± 0.21	0.31 ± 0.21	0.15 ± 0.17	0.02 ± 0.01

**Table 5.** Associated accuracy of each attribution for the Chinese and Western groups in Experiment 2 (*M* ± s.d.).

		implicit attribution			explicit attribution	
		guess	intuition	familiarity	recollection	rule
rule	Chinese	0.53 ± 0.17	0.60 ± 0.08	0.70 ± 0.16	0.69 ± 0.09	
	Western	0.47 ± 0.17	0.57 ± 0.12	0.54 ± 0.12	0.51 ± 0.19	0.50 ± 0.25
chunk	Chinese	0.56 ± 0.13	0.53 ± 0.10	0.52 ± 0.14	0.19 ± 0.09	
	Western	0.48 ± 0.17	0.54 ± 0.10	0.54 ± 0.15	0.58 ± 0.26	0.78 ± 0.05

For implicit attributions, Chinese people again showed that classification performance based on each of rules and chunks was above chance (rule: *t*_16_ = 9.58, *p* < 0.001, *d* = 2.32, *B*_H(0, 5%)_ = 66.41 × 10^14^; chunks: *t*_16_ = 2.56, *p* = 0.021, *d* = 0.62, *B*_H(0, 5%)_ = 6.70, figure 3*b*) and classification performance based on rules was greater than that based on chunks (*t*_16_ = 4.39, *p* < 0.001, *d* = 1.06, *B*_H(0, 5%)_ = 953.28). For the Western participants, classification performance based on chunks was above chance (*t*_17_ = 3.27, *p* = 0.004, *d* = 0.77, *B*_H(0, 5%)_ = 42.07), whereas for rules the evidence was not sensitive as to whether or not classification performance was above chance (*t*_17_ = 1.92, *p* = 0.072, *d* = 0.45, *B*_H(0, 5%)_ = 2.58). There was insensitive evidence as to whether or not there was a chunking advantage (*t*_17_ = 0.82, *p* > 0.250, *d* = 0.19, *B*_H(0, 5%)_ = 0.73). Crucially, the global advantage for Chinese people was greater than that for Western (*t*_33_ = 3.60, *p* = 0.001, *d* = 1.22, *B*_H(0, 5%)_ = 153.32).

### 3.3. Discussion

The purpose of Experiment 2 was to investigate whether the sort of unconscious knowledge acquired is affected by cross-cultural differences (i.e. global versus analytic differences). Chinese participants acquired unconscious knowledge of the rule and chunks, while Western participants acquired unconscious knowledge of chunks with the evidence for acquisition of the rule being insensitive. Crucially, the global advantage for Chinese people was greater than that for Westerners. That is, cross-cultural differences affected the type of unconscious knowledge people acquire. However, given that most of the participants in Experiment 2 had learnt Tang poetry at school and they may have thus been previously familiar with the inversion rule of ping–ze that we used in the artificial poetry, the question is raised of whether the differences in linguistic experience in the use of the rule in Tang poetry induce the differences in preference for global versus local processing for these particular stimuli. Experiment 3 addressed this issue by employing the inversion rule with two syllables (e.g. syllable ‘you’ in the first half maps to syllable ‘guo’ in the second half in the same corresponding position in the sequence, and syllable ‘guo’ likewise maps to syllable ‘you’ in corresponding positions, resulting to five you–guo pairs), which was familiar to neither Chinese people nor Western people, replacing the ping–ze inversion rule used in Experiment 2.

## 4. Experiment 3

The aim of Experiment 3 was to investigate whether cultural differences in the sort of unconscious knowledge acquired found in Experiment 2 were due to the differences in linguistic experience with particularly the tonal pattern of poetry. Therefore, in Experiment 3, exactly the same procedure as Experiment 2 was followed except that the inversion rule was over Chinese syllables and the chunks were of Chinese tones.

### 4.1. Method

#### 4.1.1. Participants

Seventeen Chinese students (seven females, aged from 19 to 27, *M* = 21.41; s.d. = 2.50) and 16 international Western students (11 Americans, one Canadian, two British, one French, one New Zealand; four females, aged from 19 to 29, *M* = 21.13; s.d. = 2.28) at the East China Normal University participated in this experiment and received course credits or monetary compensation for their participation. Western participants were students who came to China for a short-term exchange and learn Chinese. All had acquired mastery of the tonal syllables used in Experiment 3 to an equivalent level as the Chinese, as we show below (in the pre-test). All Chinese were China-born college students. Participants from the two groups were matched on age and gender. None of them had a history of hearing difficulties.

#### 4.1.2. Materials

**Figure 4.**
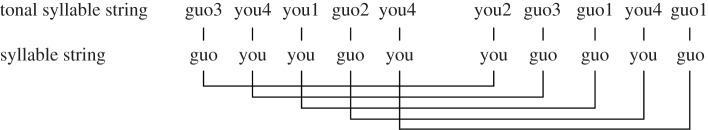
An example of grammatical strings in Experiment 3. Unlike Experiments 1 and 2, the inversion rule used in Experiment 3 was defined over the syllables of Chinese language. Specifically, the syllables (‘guo’ or ‘you’) of the first five tonal syllables of a string predicted those of the last five by an inversion relation (i.e. the syllable ‘guo’ mapped to the syllable ‘you’). For example, if the syllable of the first tonal syllable was ‘guo’, then the syllable of the sixth tonal syllable was ‘you’, and if the syllable of the second tonal syllable was ‘guo’, the tone type of the seventh tonal syllable was ‘you’, and so on.

Two Chinese syllables were selected: ‘guo’ and ‘you’, each of which was presented with tones 1–4. Thus, a total of eight tonal syllables (i.e. guo1, guo2, guo3, guo4, you1, you2, you3 and you4) were created by the same way as Experiment 1. Each string consisted of 10 tonal syllables in which the syllable type (‘you’ or ‘guo’) of the first half tonal syllables predicted the syllable type of the last half according to the inversion rule, resulting in five guo–you pairs (figure 4).

Training tonal syllable strings used in Experiment 3 were generated by replacing the tonal syllable strings used in Experiment 2. The method was as following: tone 1 and tone 4 (i.e. ping and ze) in the strings of Experiment 2 were replaced with syllables ‘you’ and ‘guo’, respectively; syllables ‘guo’, ‘you’, ‘hui’ and ‘ju’ in the strings of Experiment 2 were replaced with tone 1, tone 2, tone 3 and tone 4, respectively. For example, the training tonal syllable string of Experiment 2 ‘hui4 ju1 guo1 you4 ju1 – you1 hui4 guo4 ju1 hui4’, according to the above replacement rules, was substituted with the training string used in Experiment 3 ‘guo3 you4 you1 guo2 you4 – you2 guo3 guo1 you4 guo1’. The test tonal syllable strings used in Experiment 3 were created in the same way. Thus, there were four types of test tonal syllable strings: GH, GL, UGH and UGL based on the GACS in terms of tone 4.

#### 4.1.3. Procedure

The procedure was identical to that used in Experiment 2.

### 4.2. Results

#### 4.2.1. Pre-test

The proportions of correct response for Chinese and Western groups in the pre-test were 0.99 (s.d. = 0.02) and 0.99 (s.d. = 0.01), respectively. No difference between Chinese and Western groups was found (*t*_31_ = 0.69, *p* > 0.250, *B*_H(0, 5%)_ = 0.07).

#### 4.2.2. Test

Similarly to the previous two experiments, the classification accuracy was calculated by (*N*_C_ + 0.5)/(*N* + 1) (*N*_C_ being the number of correct responses and *N* being the total number of responses).

##### 4.2.2.1. Overall classification accuracy

**Figure 5.**
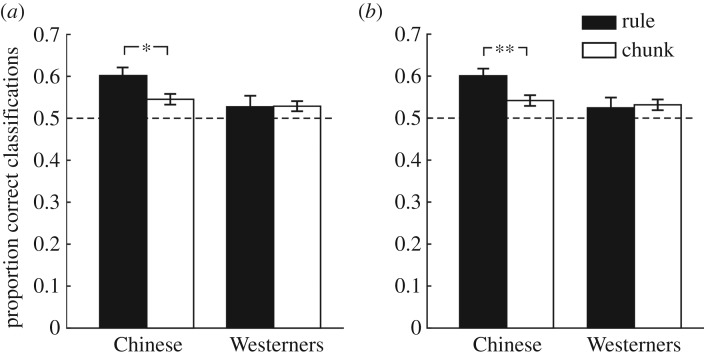
(*a*) Overall performance in Experiment 3. (*b*) Proportion correct classifications by implicit attributions in Experiment 3. Error bars indicate standard error of the mean (*p < 0.05, **p < 0.01).

For the Chinese participants, classification performance based on rule and chunks was each above chance (rule: *t*_16_ = 5.13, *p* < 0.001, *d* = 1.25, *B*_H(0, 5%)_ = 11047.37; chunks: *t*_16_ = 3.59, *p* = 0.002, *d* = 0.87, *B*_H(0, 5%)_ = 88.81, figure 5*a*) and classification performance based on the rule was greater than that based on chunks (*t*_16_ = 2.86, *p* = 0.011, *d* = 0.69, *B*_H(0, 5%)_ = 15.18), indicating a global advantage. For the Western participants, there was evidence that the classification performance based on chunks was above chance (*t*_15_ = 2.39, *p* = 0.030, *d* = 0.60, *B*_H(0, 5%)_ = 4.74), whereas the evidence was not sensitive as to whether or not classification performance for the inversion rule was above chance (*t*_15_ = 1.00, *p* > 0.250, *d* = 0.25, *B*_H(0, 5%)_ = 1.09). There was also insensitive evidence as to whether or not there was a chunking advantage for the Western participants (*t*_15_ = 0.07, *p* > 0.250, *d* = 0.02, *B*_H(0, 5%)_ = 0.52). Furthermore, the evidence as to whether or not there was a greater global advantage for the Chinese people than that for Western participants (*t*_31_ = 1.74, *p* = 0.091, *d* = 0.60, *B*_H(0, 5%)_ = 2.94) approached the conventional ball park figure for sensitivity (i.e. a *B* of 3).

##### 4.2.2.2. Unconscious structural knowledge

The response proportions of each attribution of each group are shown in table 6 and the associated accuracy of each group are shown in table 7. The attributions of guess, intuition and familiarity were combined as indicators of unconscious structural knowledge (implicit attributions), whereas those of memory and rule were combined as indicators of conscious structural knowledge (explicit attributions) [41]. Considering that only 10 Chinese and 10 Western participants chose explicit attributions in a few of test strings (mean proportion of explicit attributions was *M* = 0.08, s.d. = 0.11), we analysed classification accuracy based on only the implicit attributions.

**Table 6.** Response proportions of each attribution for the Chinese and Western groups in Experiment 3 (*M* ± s.d.).

	implicit attribution			explicit attribution	
	guess	intuition	familiarity	recollection	rule
Chinese	0.18 ± 0.19	0.42 ± 0.22	0.31 ± 0.22	0.04 ± 0.06	0.06 ± 0.11
Western	0.28 ± 0.29	0.41 ± 0.22	0.26 ± 0.24	0.04 ± 0.07	0.02 ± 0.04

**Table 7.** Associated accuracy of each attribution for the Chinese and Western groups in Experiment 3 (*M* ± s.d.).

		implicit attribution			explicit attribution	
		guess	intuition	familiarity	recollection	rule
rule	Chinese	0.54 ± 0.18	0.61 ± 0.09	0.61 ± 0.14	0.54 ± 0.20	0.66 ± 0.18
	Western	0.48 ± 0.19	0.54 ± 0.13	0.58 ± 0.18	0.59 ± 0.19	0.60 ± 0.31
chunk	Chinese	0.49 ± 0.17	0.55 ± 0. 07	0.59 ± 0.11	0.52 ± 0.15	0.66 ± 0.15
	Western	0.51 ± 0.16	0.54 ± 0.10	0.51 ± 0.16	0.52 ± 0.23	0.46 ± 0.20

For implicit attributions, Chinese people again showed that classification performance based on rule and chunks was each above chance (rule: *t*_16_ = 5.74, *p* < 0.001, *d* = 1.39, *B*_H(0, 5%)_ = 163493.60; chunks: *t*_16_ = 3.28, *p* = 0.005, *d* = 0.80, *B*_H(0, 5%)_ = 38.63, figure 5*b*). For the Western participants, classification performance based on chunks was above chance (*t*_15_ = 2.48, *p* = 0.025, *d* = 0.62, *B*_H(0, 5%)_ = 5.85). There was no evidence as to whether or not classification performance based on the rule was above chance (*t*_15_ = 0.99, *p* > 0.250, *d* = 0.25, *B*_H(0, 5%)_ = 1.03). Chinese again showed a global advantage (*t*_16_ = 3.06, *p* = 0.007, *d* = 0.74, *B*_H(0, 5%)_ = 23.52), but the evidence was not sensitive for Western people (*t*_15_ = 0.26, *p* > 0.250, *d* = 0.07, *B*_H(0, 5%)_ = 0.61). Crucially, the global advantage for Chinese people was greater than that for Western (*t*_31_ = 2.01, *p* = 0.053, *d* = 0.70, *B*_H(0, 5%)_ = 4.28).

### 4.3. Discussion

The aim of Experiment 3 was to investigate whether the cultural difference in the sort of acquired unconscious knowledge is due to the differences in the linguistic experience by swapping the inversion rule of tonal type for two syllables. The results showed that Chinese also revealed a global advantage, even with the inversion rule of two syllables, conceptually replicating Experiment 2. The data count against the greater global advantage for Chinese rather than Western people being due to simply the differential linguistic experience of the two cultural groups with the tonal patterns of Tang poetry. While experience with Chinese culture, including Tang poetry, may prime a general preference for a global rule, such a preference generalizes to rules never experienced before as such (in this case, inversions over syllables).

## 5. General discussion

The three experiments had two aims. First, we sought to establish whether Western people implicitly acquired the inversion rule instantiated in Chinese tonal syllables, while controlling both chunking and repetition structure (Experiment 1). Second, we sought to explore whether there is a priority for processing different types of structures (i.e. global symmetries versus local chunks) when both structures are simultaneously present and whether this process is affected by cross-cultural differences (Experiments 2 and 3). In the current study, we employed the Jiang *et al*. [16] artificial Chinese poetry paradigm, in which the grammar used allowed us to unconfound knowledge about the inversion rule from knowledge about the chunks of adjacent elements.

Experiment 1 showed that the Western group could implicitly learn to detect Chinese tonal strings that instantiated the inversion rule in a similar way as Chinese people when alternative structures that people could learn (like chunking and repetition structures) were carefully controlled. This finding extends Jiang *et al.* [16] and Li *et al.* [47] by showing that the inversion rule made out of the Chinese tonal syllables can be implicitly learned not only by Chinese people, but also by Westerners, at least those who have mastered the recognition of Chinese tones, implying that this paradigm can be effectively applied to implicit learning research beyond native Chinese participants. The present results also provide additional evidence that distinctively implicit learning can go beyond the learning of chunks of adjacent elements. In Experiments 2 and 3, we directly pitted knowledge of non-local dependencies against chunk knowledge, and found that Chinese participants implicitly acquired rule and chunk knowledge, even showing a striking rule-learning advantage, which was greater than that of Western participants. These results indicated that cross-cultural differences affected the type of unconscious knowledge people acquire.

Recently, Kiyokawa *et al.* [30] investigated directly for the first time cultural differences in implicit learning using the artificial grammar-learning paradigm with GLOCAL strings. Their study showed that cultural biases can profoundly affect the contents of unconscious knowledge and not just conscious processes (cf. also [31]). Similar to the findings of Kiyokawa *et al.* [30], the present study further demonstrated that the cultural biases modulated the type of unconscious knowledge (i.e. symmetries and chunks) people acquire. However, this study goes beyond these previous demonstrations of cross-cultural differences in unconscious cognition. In Kiyokawa *et al*. [30] and Fu *et al.* [31], culture affected the type of stimulus to which people preferentially paid attention, but the grammar learned was the same. Here, we investigated whether a deeper form of difference could emerge, namely in the type of regularities extracted, independent of the elements over which the regularities applied. For the first time, we showed that Chinese preferentially implicitly learnt global over local regularities to a greater extent than Westerners.

A wealth of studies on culture-related differences in conscious processing have demonstrated that Asians use a more global processing style, being especially sensitive to situational context, whereas Westerners take a more analytical approach, preferentially focusing on object-related information [27–29]. Chua *et al.* [28] monitored the eye movements of American and Chinese participants while they viewed pictures with a focal object (animal or non-living thing) on a realistic complex background. Results showed that in viewing natural scenes, American participants looked at the focal object sooner and longer than the Chinese participants, whereas the Chinese made more fixations to the background than did the Americans, indicating differences in attentional allocation between the cultures. Consistently, Lewis *et al.* [57] found that the cultural differences between Asian Americans and European Americans in allocation of attention to context emerge as early as 300 ms after stimulus appeared using ERPs. Reber argued that implicit learning requires some minimal levels of attention (cf. [58,59]). Consistent with the claim of Reber, research investigating the role of selective attention in implicit learning has shown that paying attention to relevant task features facilitates implicit learning (e.g. [60,61]). Thus, the difference in attention between Chinese and Westerners due to, for instance, different culture-specific experiences might lead to a different attentional weighting of the features inherent in the tonal syllable strings, which would lead to acquiring unconscious knowledge of different types of structures.

However, another possible explanation of the modulation of cultural biases on the type of unconscious knowledge people acquire is the claim that the Chinese tonal syllables may have been more familiar to the Chinese rather than Western people, and perhaps, differences in the familiarity of stimuli induce differences in preference for global versus local processing. Dienes & Longuet-Higgins [62] showed experimentally only highly selected participants with an interest in serialist music could implicitly learn to detect the symmetries in serialist music, whereas people who had no background in atonal music could not. Similarly, Scott & Dienes [63] found that familiarity with elemental components enhanced the capacity to implicitly learn relations between them using the artificial grammar-learning paradigm. However, Kiyokawa *et al.* [30] argued that the cultural difference in the contents of unconscious knowledge was not due to the differential familiarity of the sequence elements to the two cultural groups. We showed that the Westerners we recruited had acquired mastery of Chinese tones, so familiarity with the terminal elements of the grammar itself is unlikely to explain the cultural effects. Furthermore, given that Chinese and Western people have different experience with Chinese culture, including Tang poetry (which includes the types of inversion implemented in the experimental materials), the question was raised of whether the biases exhibited by two cultural groups in the current study could be induced by the different familiarity with Chinese poetry or language background. Future research should explore this point using non-linguistic stimuli, like coloured shapes or body movement or the like.

While we implemented an inversion or symmetry in the strings, participants may not have learnt the structure as a symmetry. Kuhn & Dienes [19] showed that exposure to musical inversions increased liking of inversions compared with non-inversions. Kuhn and Dienes controlled chunk strength in the materials to rule out this simple explanation. However, for the actual materials used, which were of fixed length, Kuhn & Dienes [64] showed an SRN (a connectionist network) could learn a set of long-distance associations between a tone in one position and a tone a fixed distance later. This remains a possible explanation for our materials; participants may have associated elements in position *n* with position *n* + 5. That is, subjects need not have learnt the symmetry *per se* in order to show learning on the test set. Relatedly, Remillard [65] has shown implicit learning of associations over seven items. Ling *et al.* [66] argued indirectly that the tonal poetry paradigm does involve learning a symmetry. Regardless of whether the participants represent the structure as a symmetry or a long-distance association (see [67], for further discussion), either representation involves a more global structure than that provided by contiguous letters making a chunk. Either way, we demonstrate a cross-cultural difference in implicit learning of global versus local structure. Furthermore, we thereby show there is no straightforward culture-independent measure of grammar complexity or difficulty (cf. [68]).

We established the implicit nature of the knowledge acquired with structural knowledge attributions, which are a form of subjective measures [32]. That is, we took implicit knowledge to be knowledge about which a certain form of metacognition is poor (cf. [69]). Seen as a measure of metacognition, the process dissociation procedure is also a form of subjective measures (of judgement knowledge), in that a response is given or withheld depending on whether the participant is confident that the item is grammatical or not (e.g. [38,70]). The main alternative method to establishing knowledge being implicit is objective measures, i.e. establishing that people are at chance at discriminating the presence or absence of the structure. However, the two main theoretical approaches to the conscious status of mental states, that is higher-order approaches and global workspace/integration approaches both motivate subjective measures [71]. An inability to discriminate the presence of the structure, as used in objective measures, probably involves degraded knowledge, whether it be conscious or unconscious [72]. Consistently, past implicit learning studies that have used objective measures, which involve asserting the null hypothesis, have often lacked power [73]. Degrading knowledge to the point it cannot directly express itself probably results in uninteresting levels of any sort of knowledge. In terms of defining implicit cognition in a way most likely to yield an interesting phenomenon, knowledge associated with poor metacognition can be reliably obtained (as we have found here; for a review, see [52]). Of course, subjective measures have their own weaknesses, such as participants not reporting what they actually think, that is the problem of possible bias [74]. For example, noise in expressing confidence as a verbal report will inevitably mean at the bottom of a scale that reports of guessing correspond in a few cases with thoughts of having some confidence (cf. [75]). In our case, the vast majority of trial-by-trial attributions indicated implicit knowledge; that is, implicit attributions could not be the result of a small amount of noise in measurement.

In conclusion, Western and Chinese participants implicitly acquired the inversion rule made out of the Chinese tonal syllables when alternative structures that people could learn (specifically, chunking and repetition structures) are carefully controlled with materials. When we directly pitted knowledge of non-local dependencies against chunk knowledge, cross-cultural differences affected the type of unconscious knowledge people acquired: Chinese participants implicitly acquired more rule knowledge than chunk knowledge, showing a striking global advantage, which exceeded that of Western participants. We show for the first time cross-cultural differences in the type of regularities implicitly acquired.

## Data accessibility

The data, materials and electronic supplementary material tables associated with this article are available through the Open Science Framework at https://osf.io/uby9a/ [76].
